# Innovative palliative care research in Europe: which horizons are we exploring?

**DOI:** 10.1177/26323524251368903

**Published:** 2025-09-16

**Authors:** Richard Harding, Lieve Van den Block

**Affiliations:** 1 Cicely Saunders Institute of Palliative Care, Policy & Rehabilitation, Florence Nightingale Faculty of Nursing Midwifery & Palliative Care, King’s College London, UK; 2 VUB-UGent End-of-Life Care Research Group, Vrije Universiteit Brussel, Belgium

**Keywords:** palliative care research in Europe, patient-centred, caregivers, European Commission, palliative care research agenda, equitable access, research investment, increasing palliative care needs, place-based care, palliative care needs

The palliative care needs of European populations are increasing and changing. Children and young people are surviving with increasingly complex health and social care needs. The number of “oldest old” is growing, and they are living with greater comorbidities. From its origins in cancer care, palliative care is now responding to a wide range of life-limiting and life-threatening conditions, within health systems that must deliver “place-based” care across diverse settings including the community. Rapidly evolving health technologies bring new opportunities to deliver novel interventions in a timely way. Public health palliative care interventions are developing, bringing palliative care beyond the boundaries of health and care services into communities, workplaces, and society.

Palliative care research is a relatively new field. A major development in our field has been the emergence of funding streams that recognize the importance of dedicated resources to discover and implement evidence-based care for patients with life-limiting and life-threatening conditions. It is known that all those who would benefit from palliative care do not currently receive it, and that without advances in delivery of effective services, this gap will widen as the population in need grows. This has enormous negative impact on health systems and the associated costs, on services, on communities, and on patients and their families.

The European Commission has established worldwide leadership in response to the need for dedicated research evidence and capacity. It has consistently developed and delivered funding calls that are either relevant to, or specifically targeted at, palliative care. This has achieved an enormous impact on our field. The breadth of carefully targeted investment has achieved considerable growth in capacity including individual career growth, in cross-national partnerships, in methodological advancement, and in the evidence base for diverse patient populations and settings. In an increasingly competitive funding landscape, dedicated funding is crucial for palliative care which is a neglected (and often undervalued) yet essential health service that affects everyone.

In this special issue “Palliative care research in Europe: which horizons are we exploring,” we have curated a collection of palliative care research studies that represent the diversity of EU-funded cutting-edge and impactful research. In alignment with the EU Cancer Mission, several projects have focused on palliative care for people with cancer, from young to older people, but across the studies, a wide range of disease populations are included. Many target the patient, but several also include a focus on caregivers, an essential part of palliative care. Jointly, they cover a wide range of countries within and beyond the EU, and span a multitude of settings including home, hospitals, communities, and even workplaces. Robust methods are used for the development, adaptation, implementation, and evaluation of interventions and programs, and new methodological approaches are being developed to improve our understanding of palliative care and people’s end of life. The projects also showcase the remarkable diversity of partnerships supporting the implementation and impact of these EU-funded projects, cutting across multiple disciplines, sectors, and health systems.

The studies demonstrate transformative European science – and the community of multidisciplinary researchers that are developing and conducting it – responding to the urgent palliative care needs of European citizens. It also demonstrates the impact of research funding that understands the importance and impact of palliative care research, and the significant ongoing impact of European Commission commitment to its citizen’s health to the end of their lives. Lastly, we note that despite the breadth of research reported in this special issue, many problems remain unresolved for patients and families. The palliative care research agenda is broad and many questions and remain unanswered. To achieve equitable access high-quality palliative care for the growing number of European citizens who need it, will require sustained research investment.

